# Iron oxide nano-adsorbent doped with nickel and palladium for phosphorus removal from water†

**DOI:** 10.1039/d5ra02256h

**Published:** 2025-07-23

**Authors:** Pamela Sepúlveda, Jonathan Suazo-Hernández, Lizethly Cáceres-Jensen, María de la Luz Mora, Juliano Denardin, Alejandra García-García, Pablo Cornejo, Binoy Sarkar

**Affiliations:** a Centro de Nanotecnología Aplicada (CNAP), Facultad de Ciencias, Ingeniería y Tecnología, Universidad Mayor Camino la Pirámide 5750 Huechuraba 8580745 Santiago Chile; b Escuela de Ingeniería en Medio Ambiente y Sustentabilidad, Facultad de Ciencias, Ingeniería y Tecnología, Universidad Mayor Camino la Pirámide 5750 Huechuraba 8580745 Santiago Chile; c Center of Plant, Soil Interaction and Natural Resources Biotechnology, Scientific and Biotechnological Bioresource Nucleus (BIOREN-UFRO), Universidad de La Frontera Avenida Francisco Salazar Temuco 01145 Chile; d Facultad de Medicina Veterinaria y Agronomía, Universidad de Las Américas Sede Concepción Chile jsuazo@udla.cl; e Future Industries Institute, University of South Australia Mawson Lakes SA 5095 Australia binoy.sarkar@unisa.edu.a; f Physical & Analytical Chemistry Laboratory (PachemLab), Nucleus of Computational Thinking and Education for Sustainable Development (NuCES), Center for Research in Education (CIE-UMCE), Department of Chemistry, Metropolitan University of Educational Sciences Santiago 776019 Chile; g University of Santiago of Chile (USACH), Center for the Development of Nanoscience and Nanotechnology (CEDENNA), Faculty of Science, Physics Department Avenida Libertador Bernardo O'higgins Santiago 3363 Chile; h Centro de Investigación en Materiales Avanzados, S.C. (CIMAV) Subsede Monterrey, Av. Alianza Norte 202, Parque PIIT Apodaca Nuevo León CP 66628 Mexico; i Centro de Estudios Avanzados en Fruticultura (CEAF) Rengo 2940000 Santiago Chile; j Centro Tecnológico de Suelos y Cultivos (CTSyC), Facultad de Ciencias Agrarias, Universidad de Talca Talca 3460000 Chile

## Abstract

Excessive phosphorus (P) in surface and ground water can cause serious environmental issues. This study aims to synthesize and characterize novel iron oxides (Fe_*x*_O_*y*_) nanoparticles (NPs) with and without Ni and Ni–Pd doping and unravel the NPs' performance and mechanism for P removal from water. X-ray diffraction, energy dispersive X-ray spectroscopy and X-ray photoelectron spectroscopy results confirmed successful doping of Ni and Ni–Pd on Fe_*x*_O_*y*_ NPs. Fe_*x*_O_*y*_–Ni NPs exhibited a higher specific surface area and isoelectric point than Fe_*x*_O_*y*_ and Fe_*x*_O_*y*_–Ni–Pd NPs. The kinetic data for P adsorption on Fe_*x*_O_*y*_ NPs fitted to the pseudo-first order model and Fe_*x*_O_*y*_–Ni and Fe_*x*_O_*y*_–Ni–Pd NPs fitted to the pseudo-second order model. Adsorption isotherm data for Fe_*x*_O_*y*_ NPs fitted to the Freundlich model and Fe_*x*_O_*y*_–Ni and Fe_*x*_O_*y*_–Ni–Pd NPs fitted to the Langmuir model. The maximum P adsorption capacity was the highest for Fe_*x*_O_*y*_–Ni (35.66 mg g^−1^) followed by Fe_*x*_O_*y*_–Ni–Pd (30.73 mg g^−1^) and Fe_*x*_O_*y*_ NPs (21.97 mg g^−1^), which was opposite to the P desorption order of these adsorbents. The adsorption and characterization analysis suggested that inner-sphere complexes and co-precipitation were the key mechanisms for P adsorption on Fe_*x*_O_*y*_–Ni and Fe_*x*_O_*y*_–Ni–Pd NPs. Therefore, Fe_*x*_O_*y*_–Ni NPs were a highly effective adsorbent for removing P from water.

## Abstract 2

Iron oxide nanoparticles doped with nickel is more efficient to remove phosphorus from water than nickel–palladium doped and undoped nano-adsorbents.

## Graphical abstract

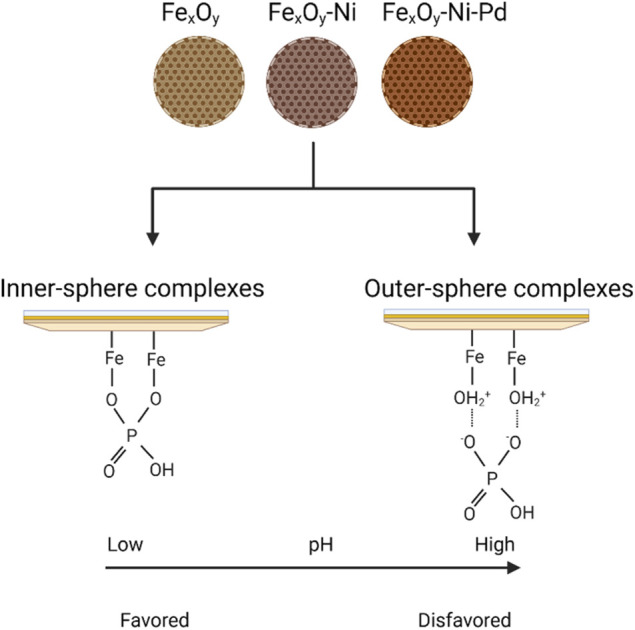

## 1. Introduction

Phosphorus (P) is an essential element for the development of life on the planet and is widely used in agriculture (fertilizers) and the chemical industry (metal preservatives and detergents).^1^ In particular, the application of P fertilizers (*e.g.*, triple superphosphate, diammonium phosphate and phosphate rock) enables P to reach the soil as di-, mono- and tri-hydrogen phosphates (H_2_PO_4_^−^, HPO_4_^2−^, and PO_4_^3−^) facilitating plant uptake and optimal plant growth.^2^ The volume and number of P fertilizers added to soils have risen in recent decades due to the increased demand for food production resulting from the world population growth.^3^ Incremental application volume and improper soil management practices have made plants unable to uptake and utilize all the added P.^4^ As a result, P is transported to aquatic systems by surface run-offs and underground water flows,^1,5^ accumulating in water bodies and producing freshwater eutrophication.^6^ Additional P inputs, especially organic and particulate P species, in water bodies from land application of composts and manures make the eutrophication situation even worse.^7^ The eutrophication of water bodies is associated with the formation of harmful algal blooms,^8^ putting to disruptions of aquatic ecosystems, which entails serious ecological and economic damage. Due to the serious impact of eutrophication, the United States Environmental Protection Agency (USEPA) has recommended that total P concentration in lake and dam water should not exceed 0.01 mg L^−1^.^9,10^ Whilst the above is an example of a regulatory measure implemented in the USA, eutrophication due to excess P in water has been a global issue in recent years as reports are emerging in many developed and developing countries.^11^ Consequently, there is a need to implement methods that can effectively reduce the concentration of P in the aquatic system.

Various simple and complex methods have been used to remove P from aquatic systems, including biological methods and chemical methods such as ion coagulation-sedimentation, exchange, electrochemical methods, and adsorption.^9,12^ In particular, adsorption is a low-cost method and reduces concentrations of various contaminants in water systems using adsorbent materials.^13^ One key characteristic to consider when using adsorbent materials to remove water contaminants is the amount of contaminant removed by a unit adsorbent mass. This is due to adsorption being a surface reaction because a high specific surface area favors the adsorption capacity of an adsorbent and a high surface area is likely to have a greater number of active sites for adsorption.^14^ Additionally, the isoelectric point (IEP) values of adsorbents directly affect the interaction with the contaminants. From IEP, it is possible to establish whether or not there are electrostatic interactions that favor adsorption, or in other words, the adsorption strength.^15^

In this context, studies report that adsorbents of iron oxide (Fe_*x*_O_*y*_) nanoparticles (NPs) like maghemite (γ-Fe_2_O_3_), hematite (α-Fe_2_O_3_), goethite (α-FeOOH), magnetite (Fe_3_O_4_), and feroxyhyte (δ-FeOOH) are the most suitable materials for water purification and remediation of soil and groundwater contaminated with oxy-anions such as arsenate (AsO_4_^3−^), selenate (SeO_4_^2−^), sulphate (SO_4_^2−^), bicarbonate (HCO_3_^−^), chromate (CrO_4_^2−^), nitrate (NO_3_^−^), and especially phosphate (PO_4_^3−^).^16–18^ Iron oxide (Fe_*x*_O_*y*_) NPs are easy to synthesize, non-toxic and inexpensive. In addition, given their high surface area and IEP values (IEP vary between 6 and 9),^19^ Fe_*x*_O_*y*_ NPs have high selectivity and affinity for phosphates. However, due to their magnetic properties and van der Waals forces, individual Fe_*x*_O_*y*_ NPs show easy agglomeration and sedimentation, decreasing their P removal efficiency and preventing their application under natural conditions.^20^ As a solution to this problem, Fe_*x*_O_*y*_ has been immobilized on inorganic/organic substrates and stabilized with organic molecules and then applied for contaminants removal.^21–23^ Furthermore, Fe_*x*_O_*y*_ NPs have been doped with metallic elements such as cobalt (Co), nickel (Ni),^24^ tin (Sn),^25^ manganese (Mn),^26^ cerium (Ce),^27^ and lanthanum (La)^28^ to increase surface area of the adsorbents and improve the adsorption performance. The adsorption performance of such NPs is increased *via* additional electronic transfer between the doped metal(s) present in the structure and the main NPs.^29^ Metal doping improves Fe_*x*_O_*y*_ NPs performance by enhancing their water stability, colloidal dispersion, oxygen evolution reaction and electrocatalytic activities.^30^ As a result of these improvements, doped Fe_*x*_O_*y*_ NPs have shown a higher maximum P adsorption capacity (*q*_max_) than pure Fe_*x*_O_*y*_ NPs. For example, Lai *et al.*^28^ reported that Fe_3_O_4_–SiO_2_–La_2_O_3_ had 2.5 times higher *q*_max_ for P than Fe_3_O_4_ NPs. In similar way, Wu *et al.*^31^ determined that *q*_max_ of P for La(OH)_3_/Fe_3_O_4_ nanocomposite was 18.6 times higher than for Fe_3_O_4_ NPs. Although there are several studies on the removal of contaminants such as Cr(vi),^32^ methylene blue and methyl orange,^33^ and As(iii)^34^ from groundwater, natural water, and wastewater using metal-doped Fe_*x*_O_*y*_ NPs, only a few publications to date have reported nutrient, such as P, removal performance of this adsorbent. This study aims to synthesize Fe_*x*_O_*y*_ NPs and dope with Ni and Ni–Pd metals, characterize the NPs and determine their P adsorption performances under different experimental conditions. It was hypothesized that Fe_*x*_O_*y*_ NPs doped with metals will show a higher surface area than pristine NPs, reduce Fe corrosion from NPs in the aqueous matrix, improve the reactivity of Fe,^35^ and in consequence will have a higher adsorption capacity of P than undoped Fe_*x*_O_*y*_ NPs. This research will contribute to the knowledge base for developing new nanomaterials to remove P and other contaminants from aqueous systems.

## 2. Materials and methods

### 2.1. Chemicals and reagents

The reagents used in the studies were FeCl_3_·6H_2_O (≥99% purity), FeCl_2_·4H_2_O (99% purity), NiCl_2_·6H_2_O (99% purity), PdCl_2_ (99% purity), NaBH_4_ (98% purity), KH_2_PO_4_ (99.99% purity), NaOH (99.9% purity), HCl (99% purity), and NaCl (≥99% purity), all of analytical grade (Merck), and double-distilled and Milli-Q water.

### 2.2. Synthesis of NPs

The NPs were synthesized by chemical reduction of Fe salts with NaBH_4_ as the reducing agent following the methodology proposed by Wang *et al.*^36^ with some modifications.^37^ The Fe_*x*_O_*y*_ NPs were obtained by mixing FeCl_3_·6H_2_O and FeCl_2_·4H_2_O in a 3 : 1 ratio in Milli-Q water. With constant magnetic stirring, 50 mL of ammonia (25%) was added in drops at 80 °C for 60 minutes (min). Next, the black solid was separated from the supernatant by magnetic separation. Finally, the solid obtained was washed with Milli-Q water and dried at 105 °C for 1 hour (h) and then at 60 °C for 24 h. The Fe_*x*_O_*y*_–Ni NPs were synthesized by mixing the precursor salts of Fe (FeCl_3_·6H_2_O plus FeCl_2_·4H_2_O in a 3 : 1 ratio) and NiCl_2_·6H_2_O in a 1 : 2 ratio in Milli-Q water and stirred for 10 min for homogenization. Then, NaBH_4_ (500 mmol L^−1^) was added dropwise for the reduction reaction to happen over 1 h, after which the solid was separated from the supernatant by magnetic separation. Finally, the solid was washed in Milli-Q water and dried at 105 °C for 1 h and then at 60 °C for 24 h. The Fe_*x*_O_*y*_–Ni–Pd NPs were synthesized in a method similar to Fe_*x*_O_*y*_–Ni NPs synthesis where PdCl_2_ was also added as a precursor salt to obtain a Ni : Fe_*x*_O_*y*_ : Pd ratio of 1 : 2 : 0.25.

### 2.3. Characterization of NPs

The surface charge of different NPs was determined by measuring the zeta potential (ZP) values using a Nano ZS instrument (Malvern Instruments, Worcestershire, United Kingdom). The NPs (15 mg) were suspended in 10 mL of NaCl solution (10 mmol L^−1^). The IEP value was obtained from ZP *versus* pH plots.

The specific surface area (SSA) of NPs was determined by applying the Brunauer–Emmett–Teller (BET) theory and the average pore diameter and pore volume applying the Barrett–Joyner–Halenda (BJH) theory following conducting N_2_ adsorption–desorption experiments at liquid N temperature on a Quantachrome Nova 1000e gas sorption analyzer (Boynton Beach, FL, USA). For each ENPs, about 0.5 g of dry powder was outgassed for about 15 h at 150 °C (7 × 10^−6^ atm) before performing the measurement.^38^

The morphology and elemental composition of the NPs were visualized using a scanning electron microscope (SEM) Zeiss EVO MA10 (Germany), working at 20 kV and energy-dispersive X-ray spectroscopy (EDS) characterizations were done with an Oxford Aztec Energy with X-act detector. Transmission electron microscope (TEM; Hitachi HT7700, Japan) images were taken with high-resolution and high-contrast visualization configurations. Additionally, NPs were characterized by X-ray diffraction (XRD) using a Bruker D2 Phaser X-ray diffractometer (Germany) equipped with Co Kα radiation source. Diffraction patterns were collected at a 2*θ* range of 10–75°. X'Pert HighScore Plus software and TOPAS software were used to analyze the XRD patterns obtained before and after adsorption of P on NPs.

The surface composition of three selected NPs was also examined by X-ray photoelectron spectroscopy (XPS) on a Thermo Fisher Scientific Escalab 250Xi instrument, operated with a conventional Al Kα source. Each special region was scanned for three different zones and analyzed using Analyzer 1.20 software. High resolution spectra obtained from O 1s, Fe 2p, Ni 2p, Pd 3d were analyzed.

### 2.4. Batch adsorption/desorption studies

#### 2.4.1. Effect of adsorbent dose

The P (as H_2_PO_4_^−^) adsorption capacity of NPs was investigated by batch experiments. To study the effect of the mass of NPs on P adsorption, 20 mL of 200 mg L^−1^ P solution^39^ at pH 5.5 ± 0.2 (by adding dilute HCl or NaOH) and background electrolyte 10 mmol L^−1^ NaCl were added to 50 mL centrifuge tubes varying the NPs mass between 10 and 80 mg. The mixture was then stirred at 200 rpm for 1440 min at 20 ± 2 °C. The tubes were centrifuged at 13 000 rpm using an ultracentrifuge for 12 min and filtered through 0.22 μm syringe filters. The P concentration in the solution was determined using the molybdate blue method on a Rayleigh UV-2601 spectrophotometer (BRAIC Co. Ltd., Beijing, China).^40^ The P amount adsorbed (*q*_e_, mg g^−1^) onto NPs were determined using eqn (1).

1 *q*_e_ = (*C*_0_ − *C*_*t*_)*V*/(*w*)

where, *C*_0_ is the initial concentration of P in solution (mg L^−1^), *C*_*t*_ is the equilibrium concentration of P in solution (mg L^−1^), *V* is the volume (L), and *w* is the mass (g) of the different NPs used.

#### 2.4.2. Effect of pH

The pH effect on P adsorption by different NPs was studied using 50 mg of NPs and 20 mL of 200 mg L^−1^ P stock solution of varying the initial pH values between 3.5 ± 0.2 and 10.5 ± 0.2 (by adding dilute HCl or NaOH) in a background electrolyte of 10 mmol L^−1^ NaCl. The mixture was added to 50 mL centrifuge tubes and stirred at 200 rpm for 1440 min at 20 ± 2 °C. The pH was also measured at the end of the experiment (pH_Final_). The tubes were centrifuged at 13 000 rpm for 12 min and the P concentration in the supernatant was determined as previously described.

#### 2.4.3. Kinetic adsorption

A kinetic adsorption study was conducted in similar set up as stated earlier with 50 mg of NPs and 200 mg L^−1^ P in 10 mmol L^−1^ NaCl solution at an initial pH 5.5 ± 0.2 (by adding dilute HCl or NaOH). Samples were withdrawn at time intervals between 0 and 1440 min (0, 2.5, 10, 30, 45, 60, 120, 200, 360, 720 and 1440 min) and analyzed for P concentration in supernatant, as described previously.

#### 2.4.4. Adsorption isotherm

Adsorption isotherms were obtained by running experiments with 50 mg of NPs and varying P concentrations between 0.5 and 200 mg L^−1^ (ref. 39) in 10 mmol L^−1^ NaCl solution at an initial pH 5.5 ± 0.2 (by adding dilute HCl or NaOH). Following stirring, centrifugation, and filtration, as described earlier, the final P concentration in the solution was determined.

#### 2.4.5. Desorption studies

To study P desorption from NPs, the first 50 mg of NPs and 20 mL of P solution (200 mg L^−1^) were mixed in 10 mmol L^−1^ NaCl solution at an initial pH 5.5 ± 0.2 (by adding dilute HCl or NaOH). The mixture was stirred at 200 rpm for 1440 min at 20 ± 2 °C. The final P concentration in the supernatant solution was determined following stirring, centrifugation and filtration, as described earlier. The residual solution was removed and 20 mL of fresh 10 mmol L^−1^ NaCl solution without any P at pH 5.5 ± 0.2 (by adding dilute HCl or NaOH) was added to the solid, and the suspension was stirred, as described above. The desorption cycle was repeated five times. After each desorption cycle, the mixture was centrifuged at 13 000 rpm for 12 min and the P concentration in the supernatant was determined as described previously. The P desorption percentage (%) by NPs after each cycle was calculated using eqn (2).

2 P desorption (%) = (*P*_desorbed_/*P*_adsorbed_) × 100

where, *P*_adsorbed_ (mg g^−1^) is the amount of P adsorbed by the NPs before NaCl treatment, and *P*_desorbed_ (mg g^−1^) is the amount of P desorbed by the NPs after NaCl treatment.

#### 2.4.6. Adsorption kinetics and isotherm models

We tested the experimental kinetic data using the pseudo-second order (PSO), pseudo-first order (PFO), and Elovich equations through non-linear fitting (Table 1SI†). The P adsorption isotherm equilibrium data were tested using the Freundlich and Langmuir equations through non-linear fitting (Table 2SI†).

### 2.5. Data analysis

All adsorption experiments were done in triplicate, and the results were presented as the mean value. The fitness of experimental data to the kinetic and isotherm models were tested non-linearly using the chi-square (*χ*^2^), coefficient of determination (known as *R*-squared, *r*^2^), and root mean square error (RMSE) values. The model fitting and figure drawing were done using the Origin 9.0 program.

## 3. Results and discussion

### 3.1. Characterization of NPs pre- and post-phosphorus adsorption

**Fig. 1:**
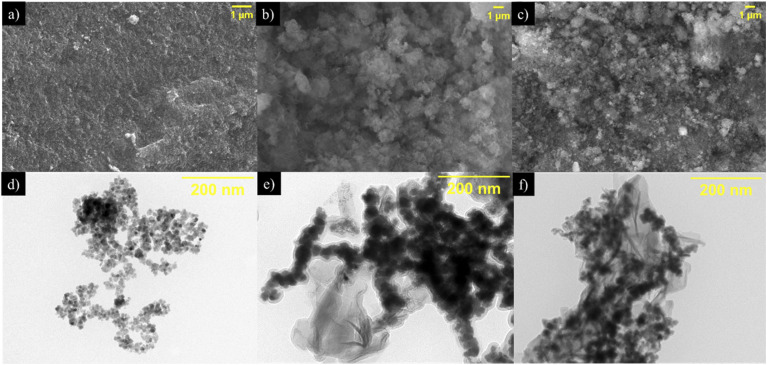
Scanning electron microscope (a–c) and transmission electron microscope (d–f) micrographs of Fe_*x*_O_*y*_ (a and d), Fe_*x*_O_*y*_–Ni (b and e) and Fe_*x*_O_*y*_–Ni–Pd (c and f) nanoparticles.

Fig. 1 shows the SEM and TEM images of the synthesized NPs. Fig. 1a shows the SEM image of Fe_*x*_O_*y*_ NPs, suggesting that SEM was unable to delineate the morphological features of these NPs due to their small size and agglomerated nature.^41,42^ Nevertheless, the TEM images in Fig. 1d confirmed a pseudo-spherical morphology of the NPs with the formation of agglomerates. An average particle size of 9.6 nm (Feret diameter) was determined for the NPs from the TEM observation. The SEM image in Fig. 1b showed the presence of two areas with different morphology of Fe_*x*_O_*y*_–Ni NPs, which was confirmed in the TEM analysis (Fig. 1e). One of these two areas is related with chains of Fe_*x*_O_*y*_ NPs with a Feret diameter of 22.6 nm and the second area is a “sheet” like morphology associated with the formation of NiO (confirmed *via* XRD and EDS Fig. 1a SI†) that coated and maintained the oxidation status of Fe_*x*_O_*y*_ NPs. The increase in size of NPs could be attributed to the NiO coating of Fe_*x*_O_*y*_ NPs. Finally, for the Fe_*x*_O_*y*_–Ni–Pd NPs, similar to the previously described case, two types of morphology were observed in SEM (Fig. 1c) and TEM (Fig. 1f) images. However, in this case, determination of the average size of NPs was not possible due to the shape and size irregularity and agglomeration of the NPs, which could indicate that Fe_*x*_O_*y*_ NPs were likely covered with amorphous layers of PdO and NiO (see EDS, Fig. 1b SI†).^43^

**Fig. 2:**
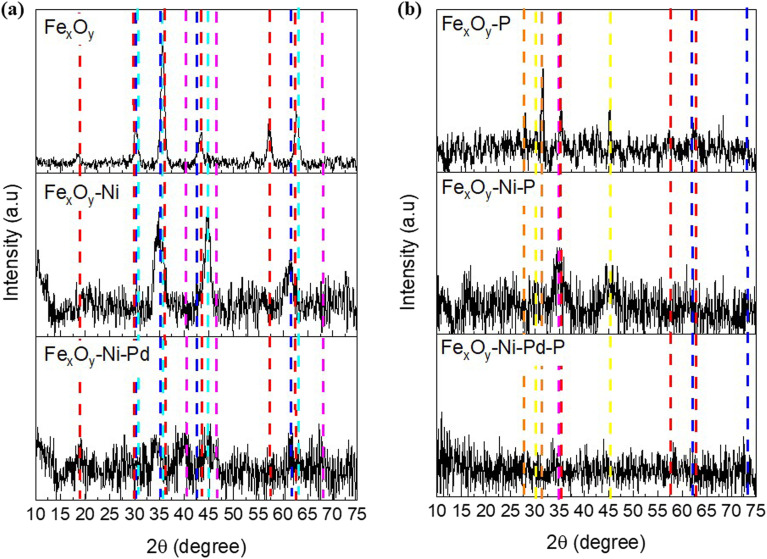
X-ray diffractograms of Fe_*x*_O_*y*_, Fe_*x*_O_*y*_–Ni and Fe_*x*_O_*y*_–Ni–Pd nanoparticles (a) before and (b) after adsorption of P. Symbols: 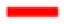 Fe_2_O_3_, 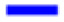 Fe_3_O_4_, 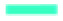 NiFe_2_O_4,_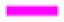 Pd, 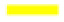 FePO_4_, 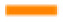 FeFe_2_(PO_4_)_2_(OH)_2_·H_2_O.

The morphological features of NPs discussed above were corroborated by XRD results (Fig. 2), where identifiable characteristic diffraction peaks of the mineral/compound phases present in the NPs (prior to P adsorption) were observed (Fig. 2a). In the diffraction pattern of Fe_*x*_O_*y*_ NPs, the characteristic peaks of Fe_2_O_3_ (ref. code: 00-004-0755) were noted at 2*θ* values of 18.4° (*h*, *k*, *l*: 111), 30.2° (*h*, *k*, *l*: 220), 35.6° (*h*, *k*, *l*: 311), 43.3° (*h*, *k*, *l*: 321), 57.3° (*h*, *k*, *l*: 511), and 62.8° (*h*, *k*, *l*: 440).^44^ The Fe_*x*_O_*y*_–Ni NPs exhibited a more amorphous phase like diffraction pattern than Fe_*x*_O_*y*_ NPs (Fig. 2a). The diffraction peaks were observed at 2*θ* values of 30.2° (*h*, *k*, *l*: 220), 35.5° (*h*, *k*, *l*: 311), 43.2° (*h*, *k*, *l*: 400) and 62.7° (*h*, *k*, *l*: 440), associated with Fe_3_O_4_ (ref. code: 01-088-0315).^45^ The second and fourth peaks (at 35.5° and 62.7° 2*θ* above) almost overlapped with characteristic peaks of NiFe_2_O_4_ [2*θ* = 30.3° (*h*, *k*, *l*: 220), 35.6° (*h*, *k*, *l*: 311), 44.8° (*h*, *k*, *l*: 400) and 63.0° (*h*, *k*, *l*: 440); ref. code: 01-074-2081]. Finally, for the XRD pattern of Fe_*x*_O_*y*_–Ni–Pd NPs (Fig. 2a), a considerably more amorphous phase like diffractogram than that of Fe_*x*_O_*y*_–Ni NPs was observed, which was consistent with the results of the SEM and TEM analyses (Fig. 1). It was possible to identify diffraction peaks associated with the presence of metallic Pd [2*θ* = 40.3° (*h*, *k*, *l*: 111), 46.8° (*h*, *k*, *l*: 200), and 68.4° (*h*, *k*, *l*: 220) (ref. code: 01-087-0645)].

The XRD patterns of NP samples following P adsorption are shown in Fig. 2b. Irrespective of the NP types, diffraction peaks were observed at 2*θ* values of 35.4° (*h*, *k*, *l*: 110), 57.3° (*h*, *k*, *l*: 018), and 62.1° (*h*, *k*, *l*: 214) associated with Fe_2_O_3_ (ref. code: 00-004-0755),^46,47^ and at 2*θ* values of 35.5° (*h*, *k*, *l*: 311), 57.1° (*h*, *k*, *l*: 511) and 62.7° (*h*, *k*, *l*: 440) associated with Fe_3_O_4_ (ref. code: 01-088-0315).^45^ New diffraction peaks suggesting the presence of P-containing phases were identified as FePO_4_ at 2*θ* values of 34.2° (*h*, *k*, *l*: 200) and 45.2° (*h*, *k*, *l*: 202) (ref. code: 00-030-0659), and FeFe_2_(PO_4_)_2_(OH)_2_·H_2_O at 2*θ* values of 28.1° (*h*, *k*, *l*: 130), 31.4° (*h*, *k*, *l*: 221) and 31.7° (*h*, *k*, *l*: 311) (ref. code: 00-026-1138), which demonstrated that adsorption of P on the NPs predominantly occurred at Fe mineral phases.

**Fig. 3:**
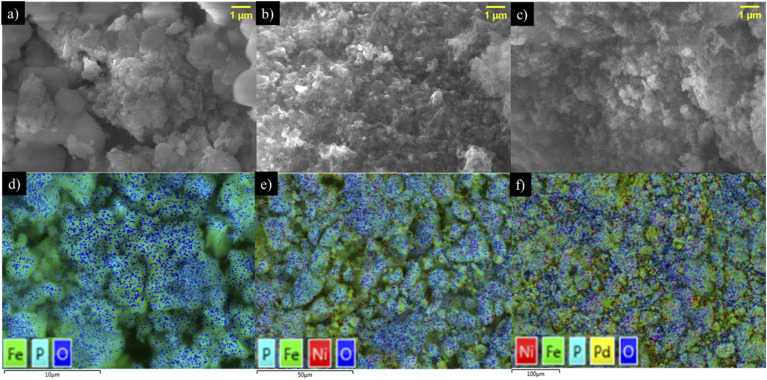
Scanning electron microscope images (a–c) and energy-dispersive X-ray spectroscopy maps (d–f) of Fe_*x*_O_*y*_ (a and d), Fe_*x*_O_*y*_–Ni (b and e) and Fe_*x*_O_*y*_–Ni–Pd (c and f) nanoparticles after adsorption of P.

The characterization using SEM-EDS following P adsorption (Fig. 3) revealed appreciable changes in the morphology of the three NPs and confirmed the presence of P in the NP structure. First, the EDS mapping confirmed the presence of Fe, Ni, and Ni–Pd respectively in Fe_*x*_O_*y*,_ Fe_*x*_O_*y*_–Ni and Fe_*x*_O_*y*_–Ni–Pd alongside other representative elements. The EDS map of Fe_*x*_O_*y*_–P (Fig. 3a and d), Fe_*x*_O_*y*_–Ni–P (Fig. 3b and e) and Fe_*x*_O_*y*_–Ni–Pd–P (Fig. 3c and f) revealed that P was mainly concentrated in the areas with the presence of O and Fe, which was consistent with the XRD results (*i.e.*, through the formation of iron phosphate (FePO_4_) phases).

**Fig. 4:**
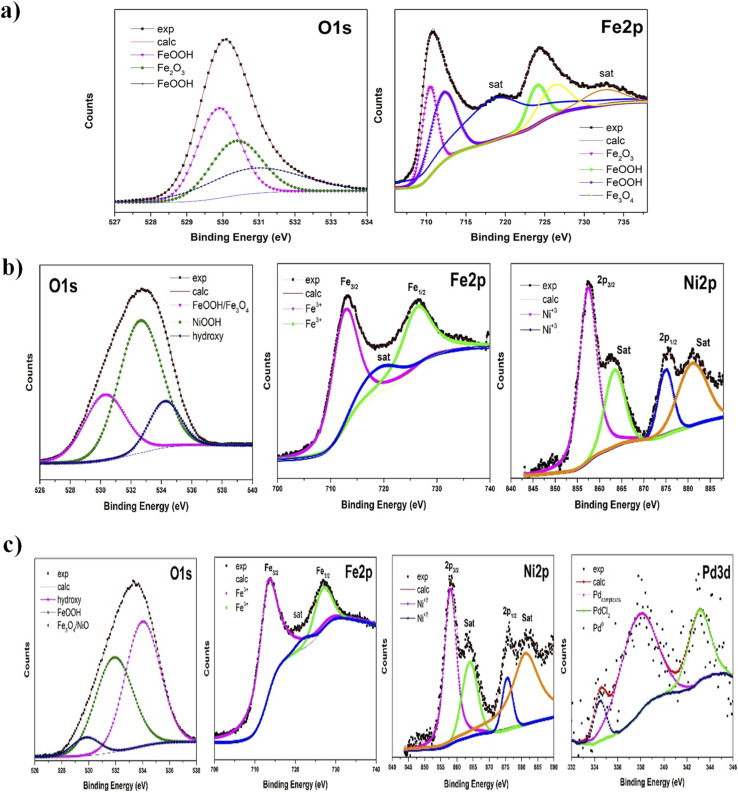
High resolution XPS spectra of (a) O 1s and Fe 2p for Fe_*x*_O_*y*_, (b) O 1s, Fe 2p and Ni 2p for Fe_*x*_O_*y*_–Ni, and (c) O 1s, Fe 2p, Ni 2p and Pd 3d for Fe_*x*_O_*y*_–Ni–Pd nanoparticles.

The XPS analysis was conducted to understand the chemical speciation of key elements in the Fe_*x*_O_*y*_, Fe_*x*_O_*y*_–Ni and Fe_*x*_O_*y*_–Ni–Pd NPs (Fig. 4). In the spectra of Fe_*x*_O_*y*_ NPs (Fig. 4a), signals corresponding to iron oxides and oxyhydroxides were identified. Table 1 displays the assigned binding energy values for the different species found, including Fe_2_O_3_ and FeOOH, as well as the possibility of existence of Fe_3_O_4_ on the surface of Fe_*x*_O_*y*_ NPs. The convolution of the high-resolution O 1s spectrum confirmed the presence of iron oxyhydroxides and oxides (iii).

**Table 1:** Binding energy values for Fe_*x*_O_*y*_, Fe_*x*_O_*y*_–Ni and Fe_*x*_O_*y*_–Ni–Pd nanoparticles

Level	Binding energy (eV)	Assignment	Reference
**Fe**_***x***_ **O**_***y***_ **NPs**			
O 1s	529.9	Fe_2_O_3_	48 and 49
	530.1	FeOOH	50
	530.4	Fe_3_O_4_	51
	531.1	FeOOH	
Fe 2p	710.5	Fe_2_O_3_	52
	712.5	FeOOH	53
	719.2	Sat.	
	724.2	FeOOH	
	726.5	Fe_3_O_4_	
	732.8	Sat.	

**Fe**_***x***_ **O**_***y***_ **–Ni NPs**			
O 1s	530.4	Fe_2_O_3_	54
	532.7	NiOOH	55
	534.2	–OOH surface hydroxy species	
Ni 2p	857.4	Ni^3+^ (2p_3/2_)	56
	863.5	Sat.	
	875.2	Ni^3+^ (2p_1/2_)	
	881.1	Sat.	
Fe 2p	713.0	Fe^3+^	57
	719.1	Sat.	
	726.5	Fe^3+^	

**Fe**_***x***_ **O**_***y***_ **–Ni–Pd NPs**			
O 1s	529.8	Fe_3_O_4_/NiO	50
	531.8	FeOOH/NiCl	58
	534.0	–OOH surface hydroxy species	55
Ni 2p	858.0	Ni^3+^ (2p_3/2_)	56
	864.0	Sat.	
	875.7	Ni^3+^ (2p_1/2_)	
	881.3	Sat.	
Fe 2p	713.6	Fe^3+^	59
	722.4	Sat.	
	727.1	Fe^3+^	
Pd 3d	334.5	Pd	60
	338.0	PdCl_4_ (Pd complexes)	
	343.2	PdCl_2_	

When Ni was added to Fe_*x*_O_*y*_ NPs (Fig. 4b), the surface chemistry of the NPs was changed, as Ni induced changes in the oxidation states of Fe, stabilizing the NPs.^56^ The result was reflected in the species found in the spectra of Fe_*x*_O_*y*_–Ni NPs. In the case of Fe 2p, a peak for Fe^3+^ species was observed compared to the spectrum of Fe_2_O_3_ sample. A shift towards higher binding energies (BE) was observed, indicating the interaction of Fe with Ni. These results are consistent with those observed by the XRD analysis results described above and previously published reports. In the Ni 2p spectrum (Fig. 4b), a shift towards higher BE was also observed, indicating an increase in the valence state of Ni due to a synergistic effect between Fe and Ni ions. The oxidation state found for Ni was 3+, indicating the presence of the chemical species NiOOH, which was also confirmed in the high-resolution O 1s spectrum (Fig. 4b). In this spectrum, the presence of FeOOH on the surface was detected, along with a peak assigned to the –OOH group. However, NiOOH was predominantly available on the surface of this material.

In the case of the Fe_*x*_O_*y*_–Ni–Pd sample (Fig. 4c), a considerable amount of FeOOH species was found, along with iron oxides such as Fe_2_O_3_ and possibly overlapped Ni oxides, seen in the peak at 529.8 eV in the high-resolution O 1s spectrum. According to the area under the curve, these oxides' quantity was smaller than iron oxyhydroxides. Only Fe^3+^ species were identified for the Fe 2p_3/2_ and Fe 2p_1/2_ peaks in the high-resolution Fe spectrum. As for Ni, the high-resolution Ni 2p spectrum revealed an oxidation state of 3+ and a shift in the corresponding signals was observed. The shift could be attributed to its interaction with Fe and Pd ions, indicating a change in the electronic nature of Ni. Lastly, in the case of the high-resolution Pd spectrum (Fig. 4c), Pd was found in the zero-valent state at a binding energy value of 334.5 eV (the similar form that was identified by XRD technique),^61^ along with Pd complexes and probable signals from the initial synthesis precursor. In this composite material, given that the amount of Fe was significantly higher than that of Ni and Pd, it was very likely that Pd and Fe were bonded. However, the presence of some Pd salts indicated that Pd did not react fully, but it affected the Ni species.

### 3.2. Adsorption study

#### 3.2.1. Effect of adsorbent dose

**Fig. 5:**
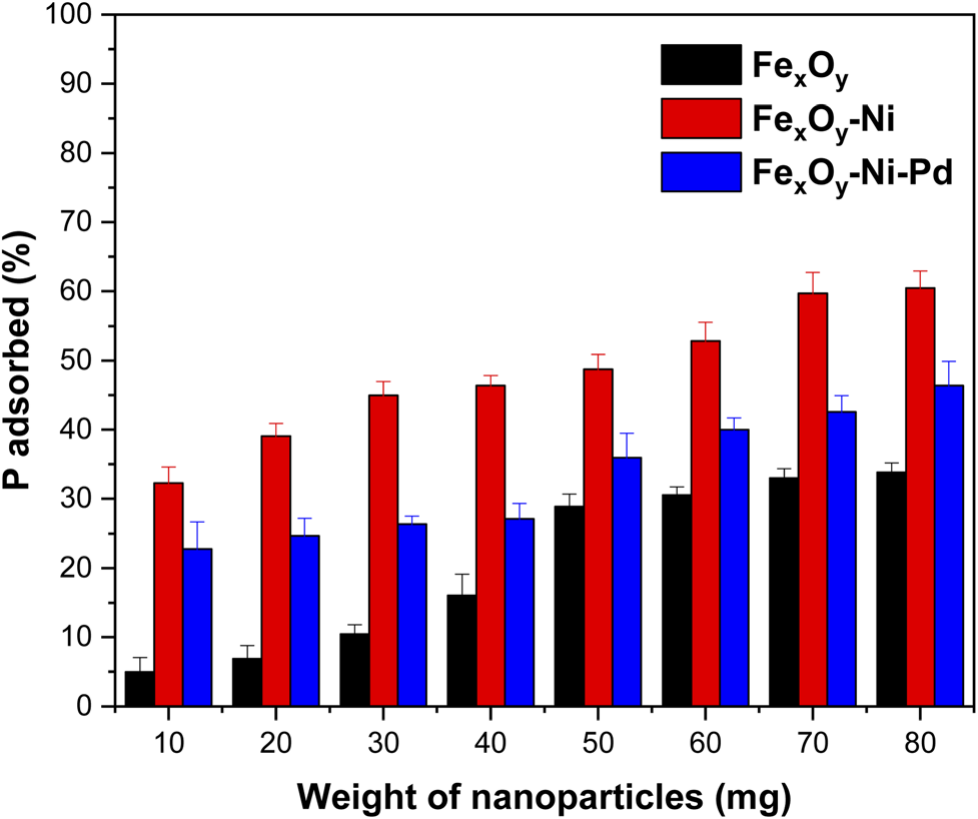
Effect of adsorbent dose on P adsorption by Fe_*x*_O_*y*_, Fe_*x*_O_*y*_–Ni, and Fe_*x*_O_*y*_–Ni–Pd nanoparticles. Initial P concentration 200 mg L^−1^ in 10 mmol L^−1^ NaCl; initial pH 5.5 ± 0.2, and reaction volume 20 mL. Error bars denote standard deviation; *n* = 3.

Fig. 5 shows the effect of the dose of three NPs on P adsorption. As the dose of the NPs increased, the removal of P increased. When the dose of NPs increased from 10 to 80 mg, the P adsorption varied between 5 and 34% for Fe_*x*_O_*y*_ NPs, between 32 and 60% for Fe_*x*_O_*y*_–Ni NPs and between 23 and 46% for Fe_*x*_O_*y*_–Ni–Pd NPs. This behavior was attributed to the adsorption of P mediated by the SSA of the NPs (Table 2), where increased SSA created a greater number of active binding sites on NPs to be occupied by P anions.^62^Fig. 5 also shows that with a mass of NPs greater than 60 mg, a plateau in P adsorption was achieved, which could be explained by the possible overlapping of reactive sites following an excessive increase of the adsorbent mass, thereby reducing the availability of the sites for adsorption. Similar results for P adsorption were obtained using a nano-α-Fe_2_O_3_/Fe_3_O_4_/biochar composite where an adsorbent dose of 50 mg was optimally considered to evaluate P adsorption capacity and rate.^63^

**Table 2:** Specific surface area, pore volume and pore diameter values of nanoparticles (NPs)

NPs	Specific surface area (m^2^ g^−1^)	Pore volume (cm^3^ g^−1^)	Pore diameter (nm)
Fe_*x*_O_*y*_	79.284	0.085	3.814
Fe_*x*_O_*y*_–Ni	113.161	0.123	3.777
Fe_*x*_O_*y*_–Ni–Pd	98.521	0.098	3.825

#### 3.2.2. Effect of pH

**Fig. 6:**
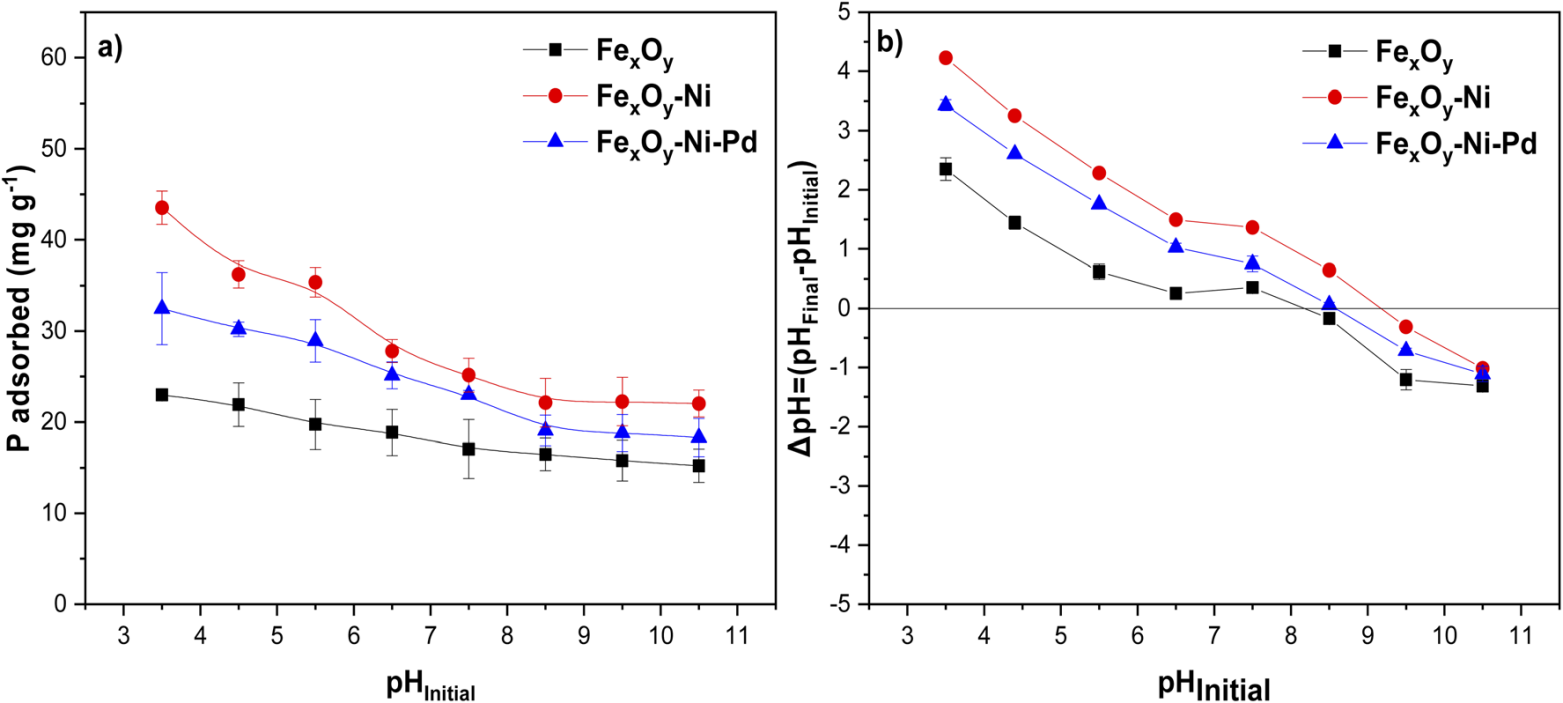
(a) Effect of initial pH on P adsorption by Fe_*x*_O_*y*_, Fe_*x*_O_*y*_–Ni, and Fe_*x*_O_*y*_–Ni–Pd nanoparticles, and (b) variation of pH (ΔpH) before and after adsorption at different initial pH values. Initial P concentration 200 mg L^−1^ in 10 mmol L^−1^ NaCl; initial pH 5.5 ± 0.2, and reaction volume 20 mL. Error bars denote standard deviation; *n* = 3.

Evaluation of the impact of solution pH on P adsorption was important because an increase in solution pH could (1) influence the dominating P species present in the solution (H_2_PO_4_^−^ and HPO_4_^2−^, p*K*_a2_ = 7.2),^64^ and increase the ionization of surface functional groups of NPs.^65^Fig. 6a illustrates that when the pH was increased from 3.5 to 10.5, the P adsorption on Fe_*x*_O_*y*_ NPs dropped from 22.98 mg g^−1^ (28.74%) to 15.22 mg g^−1^ (18.90%). This demonstrates that the pH of the solution influenced the adsorption of P by Fe_*x*_O_*y*_ NPs. The adsorption at different pH values occurred mainly through inner-sphere complex such as the bidentate phosphate complex.^66^ Conversely, an increase in solution pH demonstrated a more prominent effect on P adsorption by Fe_*x*_O_*y*_–Ni and Fe_*x*_O_*y*_–Ni–Pd NPs than by Fe_*x*_O_*y*_ NPs. For Fe_*x*_O_*y*_–Ni–Pd NPs, the P adsorption at pH 3.5 was 32.47 mg g^−1^ (39.64%), decreasing to 18.30 mg g^−1^ (22.75%) at pH 10.5. The P adsorption value for Fe_*x*_O_*y*_–Ni NPs at pH 3.5 was 43.53 mg g^−1^ (54.41%) which decreased to 22.03 mg g^−1^ (28.30%) at pH 10.5. These trends were because at a solution pH lower than the IEP of NPs, surface hydroxyl groups became protonated (–OH_2_^+^) and attracted and adsorbed the negatively charged P anions *via* inner-sphere reaction.^67^ Contrarily, at a solution pH higher than the IEP of NPs, P adsorption decreased due to electrostatic repulsion and decreased inner-sphere complex formation.^66^

**Fig. 7:**
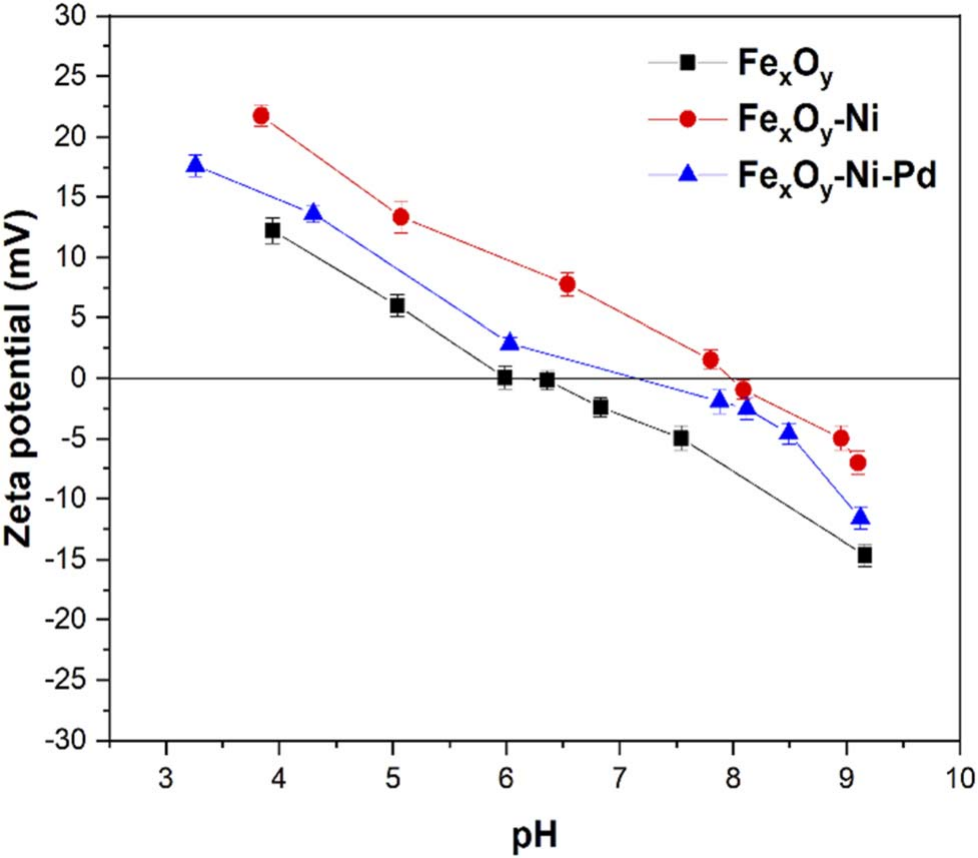
pH *versus* zeta potential (mV) curves for Fe_*x*_O_*y*_, Fe_*x*_O_*y*_–Ni, and Fe_*x*_O_*y*_–Ni–Pd nanoparticles. Error bars denote standard deviation; *n* = 3.

Additionally, OH^−^ anions present in the solution at alkaline pH could compete with the P anions, resulting in a low rate of P adsorption by NPs.^67,68^ Similar P adsorption trends by iron oxide NPs and iron oxide nanocomposites were previously reported by several researchers.^63,69,70^ The change in solution pH (ΔpH = pH_Final_ − pH_Initial_) following P adsorption on NPs supports the above discussion on the adsorption mechanism. The pH change involves the release of H^+^ or OH^−^ from NP surface functional groups and the type of the released ions could indicate the formation of inner- and outer-sphere complexes.^68,71^Fig. 6b shows that after adsorption of P on NPs the values of ΔpH were >0. In other words, OH^−^ groups were released into the solution, suggesting the formation of inner-sphere complexes between P and the NPs. At pH_Initial_ 5.5, the ΔpH value for the Fe_*x*_O_*y*_–Ni NPs was 1.41 and 3.70 times higher in relation to Fe_*x*_O_*y*_–Ni–Pd and Fe_*x*_O_*y*_ NPs, respectively, which suggested a higher affinity of Fe_*x*_O_*y*_–Ni NPs for P than the other two NPs. Therefore, the greater adsorption of P by Fe_*x*_O_*y*_–Ni NPs compared to Fe_*x*_O_*y*_–Ni–Pd and Fe_*x*_O_*y*_ NPs could be explained by the higher IEP (Fig. 7), ΔpH, and SSA (Table 2) values of Fe_*x*_O_*y*_–Ni NPs. A possible formation of oxide sheets (could be PdO; see SEM image in Fig. 3f) on Fe_*x*_O_*y*_–Ni–Pd might have hindered P adsorption to some extent. The type of metal (Ni *versus* Pd) present in the NPs could also directly affect their affinity for P, as Ogata *et al.*^72^ reported that Ni hydroxide showed a high affinity for P.

#### 3.2.3. Kinetic adsorption

**Fig. 8:**
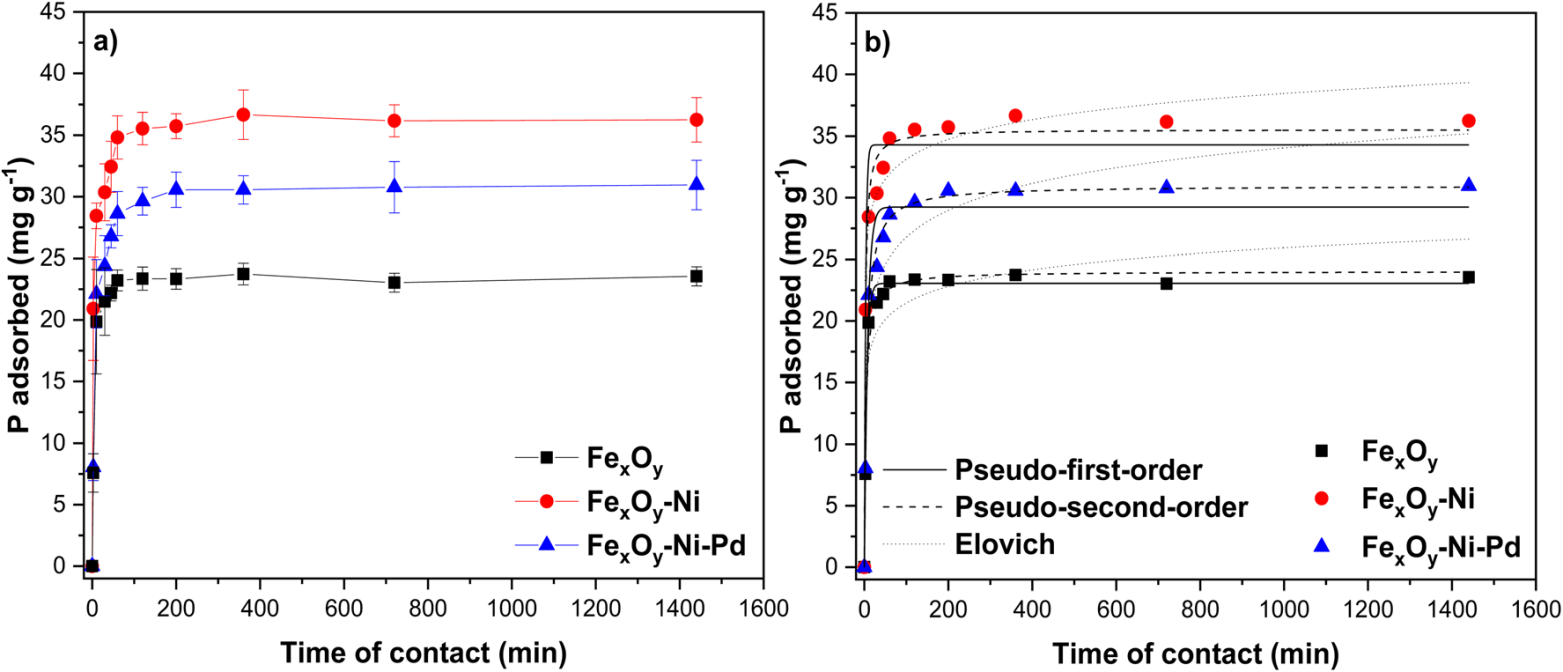
Kinetics of P adsorption on Fe_*x*_O_*y*_, Fe_*x*_O_*y*_–Ni, and Fe_*x*_O_*y*_–Ni–Pd nanoparticles (a), and model fitting lines of the experimental data (b). Initial P concentration 200 mg L^−1^ in 10 mmol L^−1^ NaCl; initial pH 5.5 ± 0.2; reaction volume 20 mL. Error bars denote standard deviation; *n* = 3.

The adsorption capacity (*q*_e_) *versus* contact time (min) plots for the adsorption of P on NPs (Fig. 8) shows that P concentration in solution decreased rapidly over time. In the first 45 min, the P adsorption capacity was high for Fe_*x*_O_*y*_, Fe_*x*_O_*y*_–Ni–Pd and Fe_*x*_O_*y*_–Ni NPs, amounting to around 22.2 mg g^−1^ (27%), 26.8 mg g^−1^ (35%) and 32.4 mg g^−1^ (41%), respectively. From 60 to 1440 min, the P adsorption capacity was practically constant, reaching a saturation (plateau of the graph) for all three NPs (Fig. 8). At the plateau stage, the adsorption capacity was 23.6 mg g^−1^ (31%), 30.9 mg g^−1^ (38%), and 36.2 mg g^−1^ (46%), respectively, for Fe_*x*_O_*y*_, Fe_*x*_O_*y*_–Ni–Pd and Fe_*x*_O_*y*_–Ni NPs. These results indicated that a long contact time would not significantly increase the efficiency of P removal using these NPs. In the first 30 min specifically, the P adsorption rate increased very fast (with a steep slope of the curves) (Fig. 8) due to (1) high attraction forces between P anions and binding sites on NPs and (2) fast diffusion of P anions on NPs to achieve a rapid equilibrium.^73^

The *q*_e_ value for P adsorption by NPs was in the order: Fe_*x*_O_*y*_–Ni > Fe_*x*_O_*y*_–Ni–Pd > Fe_*x*_O_*y*_. This trend could be explained by the smaller particle diameter of Fe_*x*_O_*y*_–Ni than Fe_*x*_O_*y*_–Ni–Pd and Fe_*x*_O_*y*_, as observed in the TEM investigation (Fig. 1b). This led to a larger SSA of Fe_*x*_O_*y*_–Ni than other two NPs (Table 2), and thus presented a greater number of adsorption sites available on the surface for adsorbing P. The incorporation of Pd on the surface of Ni-loaded NPs might have blocked the active sites, diminishing the P adsorption capacity to some extent. Previous research reported that the incorporation of a second or third metal into the structure of NPs could enhance electronic transfer between the metals present in NPs, increasing the reactivity and stability of the adsorbents in an aqueous medium.^74^ The present study did not find an increase in P adsorption following incorporation of Pd in Fe_*x*_O_*y*_–Ni; however, whether Pd incorporation affected the stability of the NPs warrants future investigation.

To understand the mechanism of P adsorption on the NPs, the kinetic P adsorption data were tested *via* fitting to the PFO, PSO and Elovich kinetic models. In the case of Fe_*x*_O_*y*_ NPs, the *r*^2^ value for PFO model was greater than the PSO model, while the *χ*^2^, and RMSE values for PFO model were lower than the obtained for PSO model (Table 3). The low *χ*^2^ value for the PFO model agreed with the similarity between the *q*_e_ determined from PFO model and that obtained from the experimental data (*q*_exp_) (Table 3), suggesting a good fit of the model. This meant that P was bound mainly on the surface of Fe_*x*_O_*y*_ NPs forming a monodentate inner-sphere complexes *via* covalent bond interaction,^75^ which corroborated with the XRD finding for the possible formation of FePO_4_ (Fig. 2b) following P adsorption. Bhattacharjee *et al.*^76^ likewise found that the PFO model correctly described P adsorption kinetics on nanoscale zero-valent iron. On the other hand, the PSO model for Fe_*x*_O_*y*_–Ni, and Fe_*x*_O_*y*_–Ni–Pd NPs presented higher *r*^2^ values (0.985 and 0.990, respectively) than those obtained from the PFO model. In addition, the *q*_e_ values from the PSO model were closer to the *q*_exp_ than the *q*_e_ values obtained from the PFO model (lower *χ*^2^ value), and the RMSE values were also lower (Table 3). Therefore, the PSO model showed a better fit of the P adsorption data on Fe_*x*_O_*y*_–Ni and Fe_*x*_O_*y*_–Ni–Pd NPs than PFO model (Table 3). This suggested that P adsorption on Fe_*x*_O_*y*_–Ni and Fe_*x*_O_*y*_–Ni–Pd NPs occurred through a chemical interaction (*i.e.*, inner-sphere complexes) between the adsorption sites and P anions forming a phosphate-iron bidentate complex,^66^ which corroborated with the ΔpH data (Fig. 6b), as explained earlier. A good fit to the PSO model also suggested that the P adsorption rate was controlled mainly by the active sites on the surface of Fe_*x*_O_*y*_–Ni NPs, where the adsorption rate was directly proportional to the number of available active sites.^77^

**Table 3:** Pseudo-first order, pseudo-second order, and Elovich model parameters for kinetic P adsorption data obtained with Fe_*x*_O_*y*_, Fe_*x*_O_*y*_–Ni, and Fe_*x*_O_*y*_–Ni–Pd nanoparticles (initial P concentration 200 mg L^−1^ in 10 mmol L^−1^ NaCl; initial pH 5.5 ± 0.2; reaction volume 20 mL)

Kinetic parameters	Fe_*x*_O_*y*_ NPs	Fe_*x*_O_*y*_–Ni NPs	Fe_*x*_O_*y*_–Ni–Pd NPs
*q*_exp_ (mg g^−1^)	23.55 ± 0.78	36.25 ± 1.21	30.95 ± 2.57
*q*_exp_ (%)	30.80	45.94	37.59

**Pseudo-first-order**			
*q*_e_ (mg g^−1^)	23.16 ± 0.26	34.30 ± 0.88	29.23 ± 0.70
*k*_1_ (×10^−3^ min^−1^)	176.74 ± 14.08	333.16 ± 68.46	127.62 ± 20.73
*r* ^2^	0.992	0.950	0.969
*χ* ^2^	0.523	6.632	3.746
RMSE	0.724	2.575	1.935

**Pseudo-second-order**			
*q*_e_ (mg g^−1^)	24.03 ± 0.46	35.55 ± 0.54	30.98 ± 0.49
*k*_2_ (×10^−3^ g mg^−1^ min^−1^)	11.18 ± 1.92	13.68 ± 2.18	5.68 ± 0.73
*h* (mg g^−1^ min^−1^)	6.46 ± 0.00	17.29 ± 0.00	5.45 ± 0.00
*r* ^2^	0.982	0.985	0.990
*χ* ^2^	1.241	1.970	1.243
RMSE	1.114	1.403	1.115

**Elovich**			
*α* (mg g^−1^ min^−1^)	1275.00 ± 91.26	35 990.63 ± 458.01	152.74 ± 17.08
*β* (g mg^−1^)	0.52 ± 0.14	0.43 ± 0.07	0.32 ± 0.06
*r* ^2^	0.857	0.966	0.899
*χ* ^2^	9.892	4.498	12.003
RMSE	3.145	2.121	3.465

Based on the PSO model, the initial adsorption rate (*h*) value for P adsorption on NPs followed the order: Fe_*x*_O_*y*_–Ni NPs > Fe_*x*_O_*y*_ NPs > Fe_*x*_O_*y*_–Ni–Pd NPs (Table 3), which suggested that Ni incorporation to NPs contributed to the generation of new chemical and/or hydrogen (H) surface groups that were available to form bonds with P anions.^78^ In addition, Fe_*x*_O_*y*_–Ni NPs showed a *h* value higher than the PSO rate constant (*k*_2_) (Table 3), indicating that at the initial stage, the available surface sites on Fe_*x*_O_*y*_–Ni NPs were quickly covered by P. Due to the high availability of adsorption sites on Fe_*x*_O_*y*_–Ni NPs, there was an increase in the concentration gradient between P in solution and P in the solid phase (on the adsorbent),^79^ which facilitated overall high P adsorption by the Ni-loaded NPs.

A chemisorption process could also be described from the moderate level fitting (*r*^2^ = 0.966; *χ*^2^ = 4.498; and RMSE = 2.121) of P adsorption data for Fe_*x*_O_*y*_–Ni NPs to the Elovich model (Table 3), where *α* is a constant related with the initial adsorption rate and *β* with the number of sites available for P adsorption.^80^ The surface of Fe_*x*_O_*y*_–Ni NPs showed a high degree of heterogeneity (*β* = 0.43 ± 0.07 g mg^−1^) with a moderate fitting to the Elovich model (Table 3), which again supported the viability of using Fe_*x*_O_*y*_–Ni NPs for an efficient P adsorption process.

#### 3.2.4. Adsorption isotherm

**Fig. 9:**
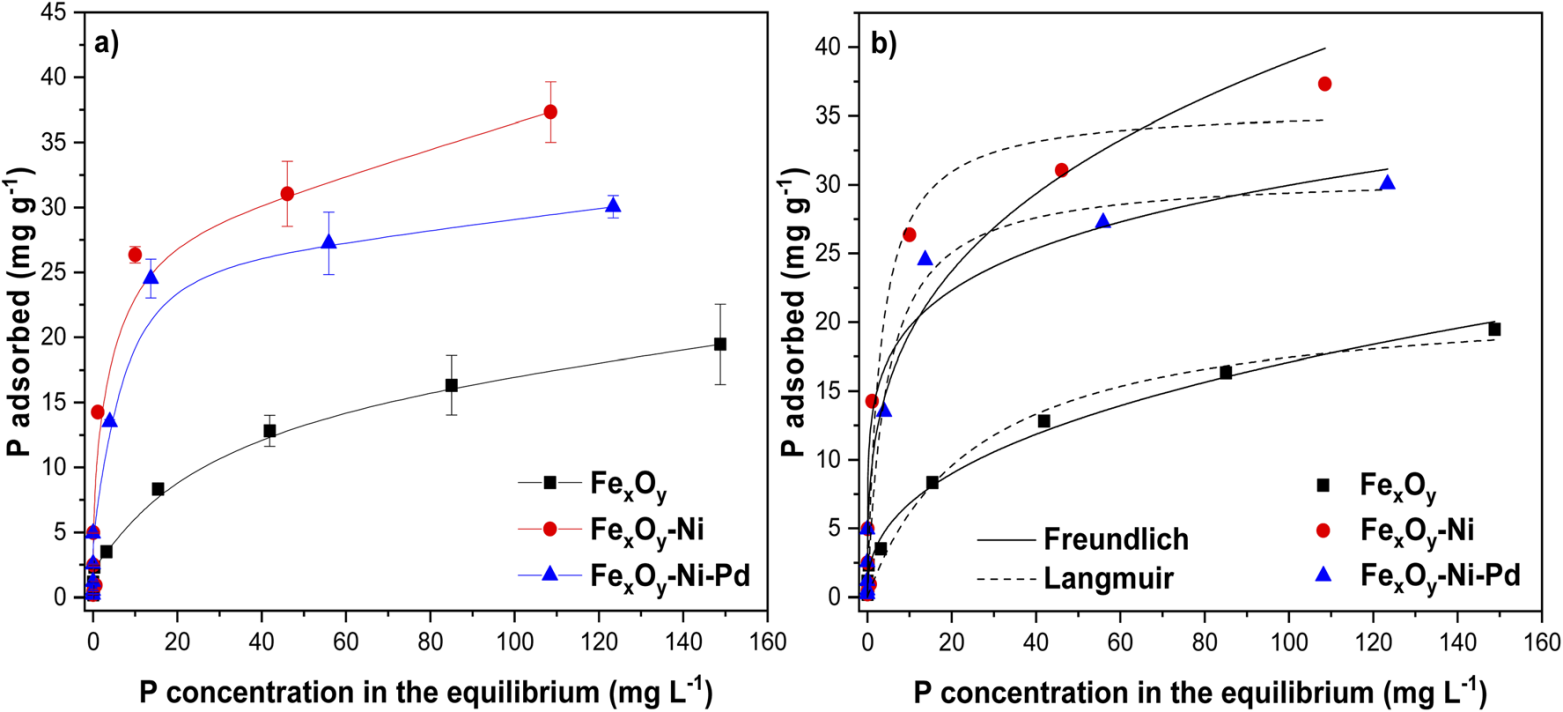
Isotherms of P adsorption on Fe_*x*_O_*y*_, Fe_*x*_O_*y*_–Ni, and Fe_*x*_O_*y*_–Ni–Pd nanoparticles at an initial pH 5.5 ± 0.2 (a), and (b) model fitting lines of the experimental data. Error bars denote standard deviation; *n* = 3.

The plots of *q*_e_*versus C*_e_ data for P adsorption on the Fe_*x*_O_*y*_ NPs followed a L-shape isotherm. In contrast, data of Fe_*x*_O_*y*_–Ni and Fe_*x*_O_*y*_–Ni–Pd NPs followed a H-shape isotherm (Fig. 9a). These results indicated that the Fe_*x*_O_*y*_–Ni and Fe_*x*_O_*y*_–Ni–Pd NPs had a higher affinity for P than Fe_*x*_O_*y*_ NPs. In addition, the H-shape curve showed that a chemisorption mechanism controlled the adsorption of P on Fe_*x*_O_*y*_–Ni and Fe_*x*_O_*y*_–Ni–Pd NPs.^81^

The experimental data were fitted to the Langmuir and Freundlich isothermal models to determine the maximum adsorption capacity (*q*_max_) and adsorption intensity (*n*).^82^ The Freundlich model was able to describe the behavior of P adsorption on Fe_*x*_O_*y*_ NPs (Fig. 9b; Table 4), suggesting that the adsorption was a multilayer process on a heterogeneous surface. The Langmuir model particularly presented a better fit to the experimental data of Fe_*x*_O_*y*_–Ni and Fe_*x*_O_*y*_–Ni–Pd NPs than the Freundlich model (Table 4). This suggested that P adsorption on Fe_*x*_O_*y*_–Ni and Fe_*x*_O_*y*_–Ni–Pd NPs occurred through monolayer interactions on a homogeneous surface.^83^ The isotherm results thus conformed with the P adsorption mechanisms (*i.e.*, chemical interactions) on Fe_*x*_O_*y*_–Ni and Fe_*x*_O_*y*_–Ni–Pd NPs hypothesized from the kinetic modelling results, as also described previously by Wang *et al.*^84^ for P adsorption on Fe^0^/iron oxide-coated diatomite NPs.

**Table 4:** Langmuir and Freundlich isotherm model parameters for P adsorption on Fe_*x*_O_*y*_, Fe_*x*_O_*y*_–Ni, and Fe_*x*_O_*y*_–Ni–Pd nanoparticles (NPs) at initial pH 5.5 ± 0.2

Isotherm parameters	Fe_*x*_O_*y*_ NPs	Fe_*x*_O_*y*_–Ni NPs	Fe_*x*_O_*y*_–Ni–Pd NPs
**Langmuir**			
*K*_L_ (L mg^−1^)	0.04 ± 0.00	0.33 ± 0.13	0.22 ± 0.07
*q*_max_ (mg g^−1^)	21.97 ± 1.65	35.66 ± 2.74	30.73 ± 1.97
*r* ^2^	0.981	0.951	0.970
*χ* ^2^	1.163	12.07	5.26
RMSE	1.079	3.474	2.233
			
**Freundlich**			
*K*_F_ (mg g^−1^)(L mg^−1^)^1/*n*^	2.71 ± 0.30	9.43 ± 2.13	12.98 ± 2.55
*n*	2.51 ± 0.16	3.25 ± 0.59	5.46 ± 1.50
*r* ^2^	0.992	0.908	0.954
*χ* ^2^	0.514	22.86	8.12
RMSE	0.717	4.782	2.850

The *q*_max_ value for P adsorption was in the order: Fe_*x*_O_*y*_–Ni > Fe_*x*_O_*y*_–Ni–Pd > Fe_*x*_O_*y*_ NPs (Table 4). The *q*_max_ for Fe_*x*_O_*y*_–Ni NPs was 1.16 and 1.62 times higher than Fe_*x*_O_*y*_–Ni–Pd and Fe_*x*_O_*y*_ NPs, respectively. These results could be attributed to the physicochemical properties such as larger SSA, pore volume and greater IEP of Fe_*x*_O_*y*_–Ni than Fe_*x*_O_*y*_–Ni–Pd and Fe_*x*_O_*y*_ NPs (Table 2; Fig. 7). For example, Fe_*x*_O_*y*_–Ni, Fe_*x*_O_*y*_–Ni–Pd, and Fe_*x*_O_*y*_ NPs had an IEP value of 7.99, 7.16, and 6.16, respectively (Fig. 7). At a solution pH value of 5.5, the surface of Fe_*x*_O_*y*_–Ni NPs would have more positive charges than the other two NPs, promoting the adsorption of P anions. High SSA, pore volume and IEP would also contribute to a higher number of active adsorption sites available for P anions on Fe_*x*_O_*y*_–Ni NPs than Fe_*x*_O_*y*_–Ni–Pd and Fe_*x*_O_*y*_ NPs. The adsorption affinity (*K*_L_) values (Table 4) again indicated that the P-binding was more favorable on Fe_*x*_O_*y*_–Ni NPs than Fe_*x*_O_*y*_–Ni–Pd, and Fe_*x*_O_*y*_ NPs, which was in line with the high affinity of Ni to P previously reported by Ogata *et al.*^72^

Table 5 lists previously reported values of P adsorption capacity (modelled) for a number of Fe_*x*_O_*y*_ based materials with a projection for use in P removal from water. The Fe_*x*_O_*y*_–Ni NPs studied in this research performed better than or at least similar to the previously reported P adsorbents (Table 5), and in consequence these can be useful for the future development of sustainable and practical P removal technologies. The size, morphology, surface charge, SSA, and other surface characteristics of NPs alongside experimental conditions (*e.g.*, pH, and ionic strength) could considerably affect the P adsorption capacity.^85^ The current study also demonstrated that incorporating a third metal (Fe–Ni–Pd) in the structure of NPs decreased the P adsorption capacity compared to the bimetallic adsorbent (Fe–Ni) due to chemical changes on the surface of NPs.

**Table 5:** Modelled P adsorption capacity values of Fe_*x*_O_*y*_ based adsorbent materials

Adsorbents	Model used	Initial pH	Equilibrium time	Maximum adsorption capacity or relative adsorption capacity (mg g^−1^)	Reference
Fe_3_O_4_–diatomite	Langmuir	7.0	60 min	11.89	86
Fe_3_O_4_–illite clay	Langmuir	7.0	60 min	5.48	86
Fe^0^/iron oxide–diatomite	Langmuir	7.0	30 min	37.0	84
Fe_2_O_3_–halloysite nanotubes	Langmuir	4.0	4 h	5.13	87
La(OH)_3_/Fe_3_O_4_	Langmuir	7.0	2 h	83.5	31
Iron–carbon nanotubes	Langmuir	—	3 h	36.5	69
Iron(iii)–copper(ii) binary oxides	Langmuir	7.0	24 h	35.2	88
Fe_3_O_4_@mZrO_2_	Langmuir	3.0	24 h	39.1	23
Zirconium–iron oxide	Freundlich	—	24 h	21.3	89
Fe_3_O_4_–SiO_2_–La_2_O_3_	Langmuir	6.6	24 h	27.8	28
Fe–Mn oxide	Langmuir	7.0	24 h	18.4	90
Fe–Zr binary oxide	Langmuir	4.0	24 h	13.65	91
Fe_*x*_O_*y*_–Ni–Pd	Langmuir	5.5	24 h	30.73	This study
Fe_*x*_O_*y*_–Ni	Langmuir	5.5	24 h	35.66	This study

## 4. Desorption study

**Fig. 10:**
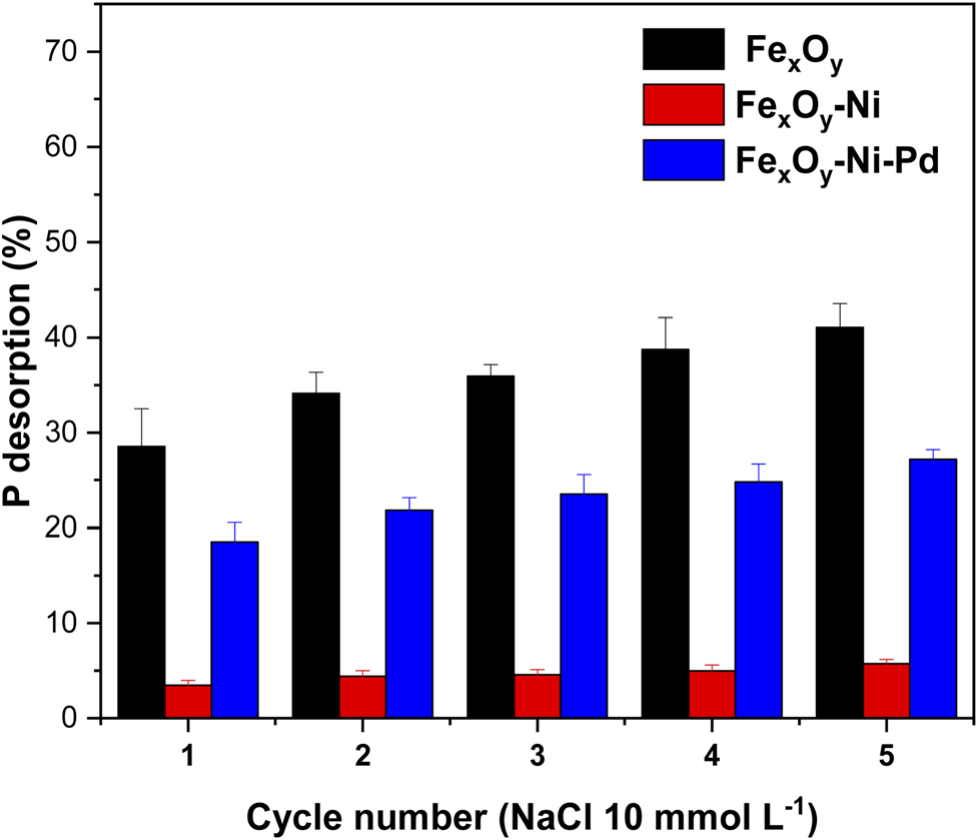
Desorption (%) of P from Fe_*x*_O_*y*_, Fe_*x*_O_*y*_–Ni, and Fe_*x*_O_*y*_–Ni–Pd nanoparticles in NaCl (10 mmol L^−1^) solution over 1440 min at 20 ± 2 °C. Error bars denote standard deviation; *n* = 3.

Desorption of P from the NPs was conducted using a NaCl solution (10 mmol L^−1^) as the extracting agent to analyze the cost-effectiveness, reusability, and stability of the NPs synthesized.^92^Fig. 10 shows that after five successive cycles, the desorption of P from Fe_*x*_O_*y*,_ Fe_*x*_O_*y*_–Ni and Fe_*x*_O_*y*_–Ni–Pd NPs reached 41.09%, 5.73%, and 27.16%, respectively. The amount of P desorbed from Fe_*x*_O_*y*_–Ni NPs was 0.14 and 0.21 times lower than Fe_*x*_O_*y*_ and Fe_*x*_O_*y*_–Ni–Pd NPs, respectively. These results are consistent with the *K*_L_ values obtained from the Langmuir isothermal model for the three NPs (Table 4). Moreover, these results reiterated that the presence of Ni helped the Fe_*x*_O_*y*_ NPs to form a stronger bond with P than the Pd–Ni mixture, confirming the formation of Fe–P complex for Fe_*x*_O_*y*_–Ni as identified by XRD analysis (Fig. 2). In terms of easy reusability and cost-effectiveness for removing anions from water, Fe_*x*_O_*y*_ NPs proved to be the most suitable. On the other hand, Fe_*x*_O_*y*_–Ni NPs would be an ideal candidate for permanently immobilizing an anionic contaminant in water and subsequent recovery from that system due to its magnetic properties.^93^

## 5. Scalability and applicability challenges

The performance of Fe_*x*_O_*y*_–Ni NPs in terms of P removal was superior to that of Fe_*x*_O_*y*_ NPs, and Fe_*x*_O_*y*_–Ni–Pd NPs in a laboratory setting. However, further studies are necessary to investigate the scalability and practical application of these NPs and to support their large-scale implementation. At the same time, various issues must be resolved before they can be used. These include the necessity to provide long-term stability, which covers several adsorption/desorption cycles under real water conditions, and the issue of NPs aggregation, which could decrease SSA and consequently their adsorption efficiency. Furthermore, research has shown that the selectivity towards P may be affected in real water systems due to the presence of competing anions, such as HCO_3_^−^, CrO_4_^2−^, AsO_4_^3^, and SO_4_^2−^, and organic matter.^87,94–96^

From an operational perspective, synthesis at an industrial scale and adherence to environmental regulations, particularly concerning the potential leaching of Fe and/or Ni, must also be considered. Therefore, an excellent alternative is to support these NPs on bulk materials or polymer substrates to reduce the Fe lixiviation process.^97,98^ Despite these limitations, the adsorption capacity and magnetic recoverability of these NPs highlight their potential for development into sustainable and effective P adsorption technology, particularly with further optimization and pilot-scale validation.

## 6. Conclusions

This study synthesized, characterized, and tested Fe_*x*_O_*y*_ nanoparticles (NPs) with and without Ni and Ni–Pd metal doping as an adsorbent for removing P from water. The maximum phosphorus (P) adsorption capacity (*q*_max_) was demonstrated by Fe_*x*_O_*y*_–Ni followed by Fe_*x*_O_*y*_–Ni–Pd, and Fe_*x*_O_*y*_ NPs. Contrarily, the desorption of P followed the order: Fe_*x*_O_*y*_ > Fe_*x*_O_*y*_–Ni–Pd > Fe_*x*_O_*y*_–Ni NPs. The NPs underwent a surface transformation process, forming new mineralogical phases corresponding to amorphous iron oxides species (FePO_4_ and FeFe_2_(PO_4_)_2_(OH)_2_·H_2_O), following the adsorption of P. The P adsorption kinetics for Fe_*x*_O_*y*_–Ni, and Fe_*x*_O_*y*_–Ni–Pd NPs was described by the pseudo-second order model, showing that the P adsorption occurred by inner-sphere bidentate complexes, and for Fe_*x*_O_*y*_ NPs, the pseudo-first order model showed a better mathematical fit. The P adsorption on Fe_*x*_O_*y*_–Ni, and Fe_*x*_O_*y*_–Ni–Pd NPs were explained by the Langmuir model, suggesting that the P adsorption occurred by chemisorption. Meanwhile, for Fe_*x*_O_*y*_ NPs, the experimental data fitted well to the Freundlich model. Overall, the results suggested that the Ni doping generated an increased specific surface area, and isoelectric point for Fe_*x*_O_*y*_ NPs, creating additional sites for P adsorption and enabling inner-sphere complexation and co-precipitation mechanisms on the adsorbent surface. However, doping with Ni–Pd mixture most likely created a PdO coating on Fe_*x*_O_*y*_ NPs partially blocking the P adsorption sites, and reducing adsorption affinity. In conclusion, multi-metal oxide nanocomposites—Fe_*x*_O_*y*_–Ni NPs was presented as an efficient adsorbent for P removal from polluted water. Future studies should investigate the specific reactions between bi- and tri-metallic NPs and P, long-term stability of NPs, and effect of parameters such as temperature and ionic strength on adsorption performance and use contaminated real wastewater samples. In addition, characterization of NPs using advanced techniques such as X-ray photoelectron spectroscopy and/or transmission electron microscopy-energy dispersive spectroscopy after P adsorption is necessary to determine the thickness and uniformity of NPs surface layers.

## Author contributions

Pamela Sepúlveda: conceptualization; funding acquisition; investigation, formal analysis; writing – original draft and review & editing. Jonathan Suazo-Hernández: investigation, formal analysis; methodology; software; conceptualization; visualization; writing – original draft; writing – review & editing. Lizethly Cáceres-Jensen: conceptualization; methodology; visualization; writing – original draft. María de la Luz Mora: conceptualization; funding acquisition. Juliano Denardin: conceptualization; funding acquisition. Alejandra García-García: methodology; resources; supervision, writing – review & editing. Pablo Cornejo: conceptualization; writing – original draft. Binoy Sarkar: conceptualization; supervision; investigation; validation; writing – review & editing.

## Conflicts of interest

The authors declare that there are no conflicts of interest.

## Supplementary Material

RA-015-D5RA02256H-s001

## Data Availability

Data will be made available on request.
