# Supplementary material for: Iron oxide nano-adsorbent doped with nickel and palladium for phosphorus removal from water
Source: RSC Adv. 2025 Jul 23;15(32):26321–37. doi: 10.1039/d5ra02256h (PMC12284633; doi:10.1039/d5ra02256h)
Supplement: RA-015-D5RA02256H-s001 [file RA-015-D5RA02256H-s001.pdf]

Supplementary Information (SI) for RSC Advances.
This journal is © The Royal Society of Chemistry 2025

Supplementary Information for:

Iron oxide nano-adsorbent doped with nickel and palladium for phosphorus removal from water

Pamela Sepúlveda[a,b], Jonathan Suazo-Hernández[c,d,e**], Lizethly Cáceres-Jensen[f], María de la Luz Mora[c], Juliano Denardin[g], Alejandra García-García[h], Pablo Cornejo[i,j], Binoy Sarkar[e*]

[a]Centro de Nanotecnología Aplicada (CNAP), Facultad de Ciencias, Ingeniería y Tecnología, Universidad Mayor. Camino la Pirámide 5750, Huechuraba 8580745, Santiago, Chile

[b]Escuela de Ingeniería en Medio Ambiente y Sustentabilidad, Facultad de Ciencias, Ingeniería y Tecnología, Universidad Mayor, Camino la Pirámide 5750, Huechuraba 8580745, Santiago. Chile

[c]Center of Plant, Soil Interaction and Natural Resources Biotechnology, Scientific and Biotechnological Bioresource Nucleus (BIOREN-UFRO), Universidad de La Frontera, Avenida Francisco Salazar 01145, Temuco, Chile

[d]Facultad de Medicina Veterinaria y Agronomía, Universidad de Las Américas, Sede Concepción, Chile

[e]Future Industries Institute, University of South Australia, Mawson Lakes, SA 5095, Australia

[f]Physical & Analytical Chemistry Laboratory (PachemLab), Nucleus of Computational Thinking and Education for Sustainable Development (NuCES), Center for Research in Education (CIE-UMCE), Department of Chemistry, Metropolitan University of Educational Sciences, Santiago 776019, Chile

[g]University of Santiago of Chile (USACH), Center for the Development of Nanoscience and Nanotechnology (CEDENNA), Faculty of Science, Physics Department. Avenida Libertador Bernardo O´higgins 3363, Santiago, Chile

[h]Centro de Investigación en Materiales Avanzados, S.C. (CIMAV), Subsede Monterrey, Av. Alianza Norte 202, Parque PIIT, Apodaca, Nuevo León, CP 66628, México

[i]Centro de Estudios Avanzados en Fruticultura (CEAF), Rengo 2940000, Santiago, Chile

[j]Centro Tecnológico de Suelos y Cultivos (CTSyC), Facultad de Ciencias Agrarias, Universidad de Talca, Talca 3460000, Chile

Corresponding authors:

[*]Future Industries Institute, University of South Australia, Mawson Lakes, SA 5095, Australia. E-mail address: binoy.sarkar@unisa.edu.a

[**]Facultad de Medicina Veterinaria y Agronomía, Universidad de Las Américas, Sede Concepción, Chile. E-mail address: jsuazo@udla.cl

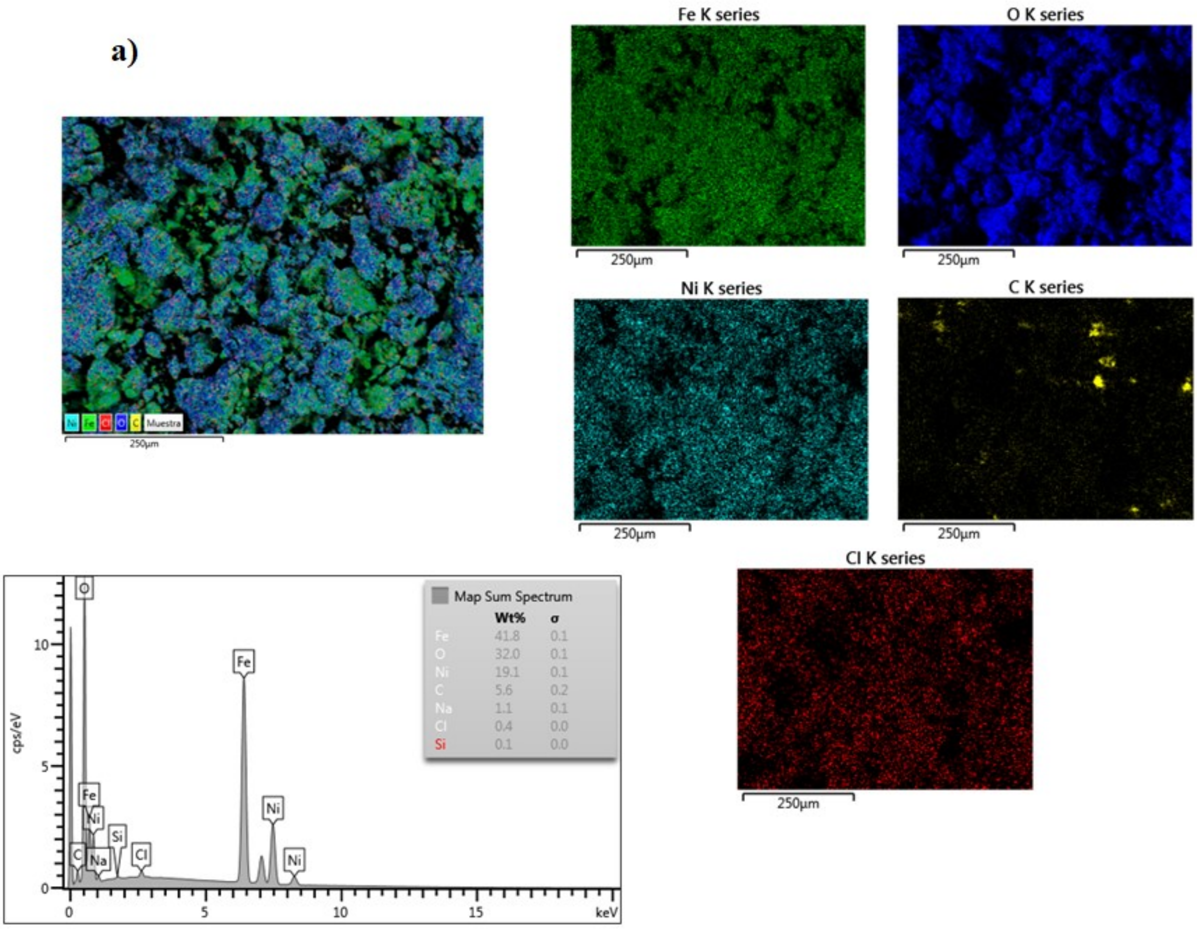

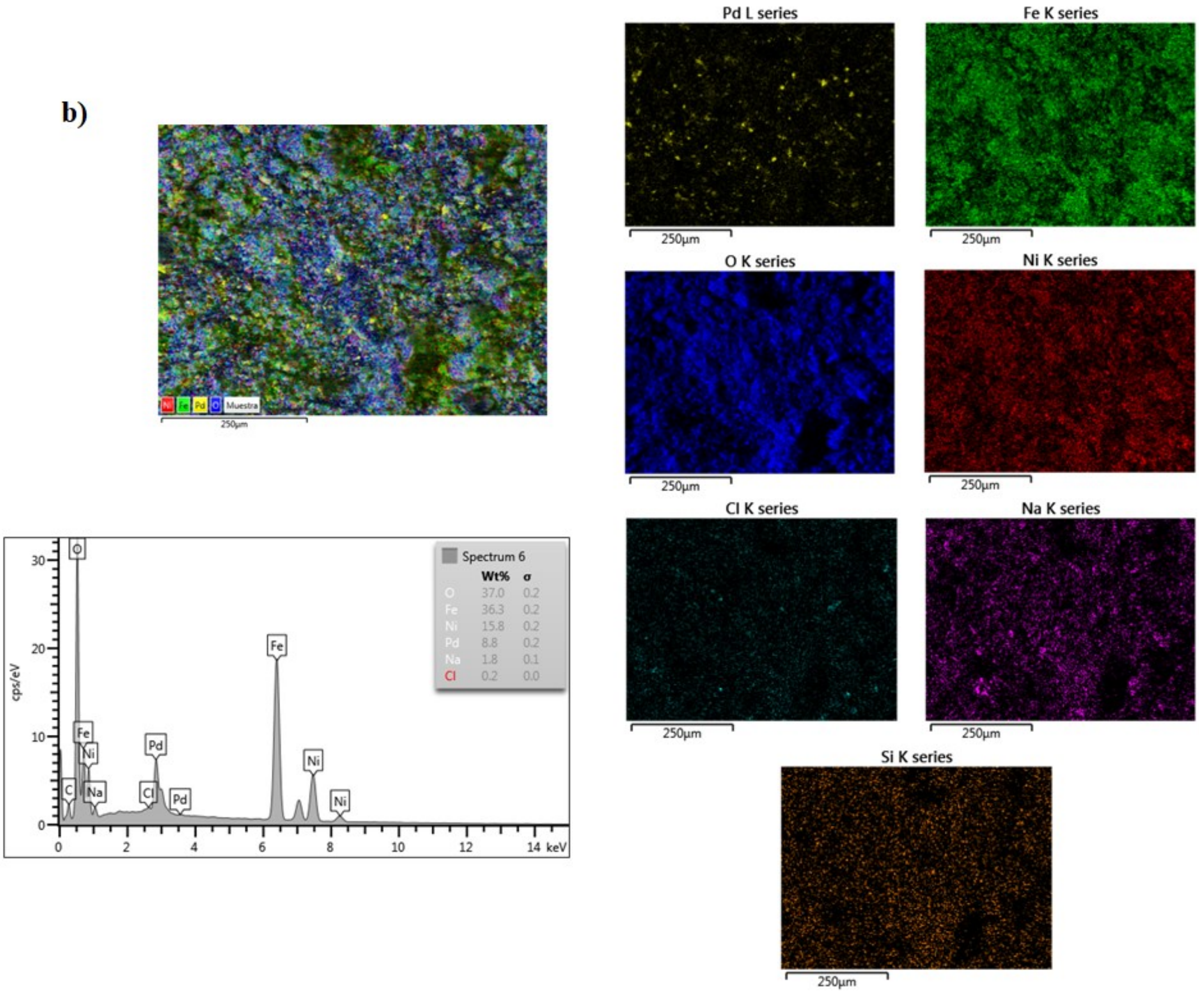

Figure 1 SI. Scanning electron microscope images coupled to energy-dispersive X-ray spectroscopy maps of FexOy-Ni (a) and FexOy-Ni-Pd (b) nanoparticles after adsorption of P.

Table 1 SI. The kinetic models used for the description of phosphorus (P) adsorption [1].

Kinetic Equations	Expression Formula	Parameters
Pseudo-first-order	$q_t = q_e(1 - e^{-k_1 t})$	q_t = Amount of P adsorbed at any time (mg g^{-1}). q_e = Amount of P adsorbed at equilibrium (mg g^{-1}). k_1 = PFO rate constant (min^{-1}). k_2 = PSO rate constant (g mg^{-1} min^{-1}). t = Time (min) α = Initial rate constant (mg g^{-1} min^{-1}). β = Number of sites available for the adsorption and desorption constant (g mg^{-1}).
Pseuso-second-order (PSO)*	$q_t = \frac{k_2 q_e^2 t}{1 + k_2 q_e t}$	
Elovich	$q_t = \frac{1}{\beta} \ln(1 + \alpha\beta t)$	

*From PSO initial adsorption rate (h), can be calculated by multiplying $k_2 q_t^2$ (mg g^{-1} min^{-1}).

Table 2 SI. The isotherm models used for the description of phosphorus (P) adsorption [1].

Isotherm Equations	Expression Formula	Parameters
Langmuir	$q_e = \frac{q_{max} K_L C_e}{1 + K_L C_e}$	q_e = Amount of P adsorbed per unit mass of the adsorbent at equilibrium (mg g^{-1}). q_{max} = Maximum adsorption capacity (mg g^{-1}). C_e = Concentration of P at equilibrium in the solution (mg L^{-1}). K_L = Constant of the adsorption energy (L mg^{-1}). K_F = Freundlich adsorption coefficient (mg g^{-1}) (L $mg^{-1})^{1/n}$. n = Adsorption intensity ($1 < n < 10$).
Freundlich	$q_e = K_F C_e^{1/n}$	

References

[1] J. Wang, G. Zhang, S. Qiao, J. Zhou, Magnetic Fe^0/iron oxide-coated diatomite as a highly efficient adsorbent for recovering phosphorus from water, Chem. Eng. J. 412 (2021) 128696, DOI: 10.1016/j.cej.2021.128696.
